# Socioeconomic disparity in the association between fine particulate matter exposure and papillary thyroid cancer

**DOI:** 10.1186/s12940-023-00972-1

**Published:** 2023-02-23

**Authors:** Philip Crepeau, Zhenyu Zhang, Rhea Udyavar, Lilah Morris-Wiseman, Shyam Biswal, Murugappan Ramanathan, Aarti Mathur

**Affiliations:** 1 Department of Surgery, Johns Hopkins University School of Medicine, Baltimore, MD USA; 2 Department of Global Health, Peking University School of Public Health, Beijing, China; 3 Institute for Global Health and Development, Peking University, Beijing, China; 4 Department of Environmental Sciences, Johns Hopkins Bloomberg School of Public Health, Baltimore, MD USA; 5 Department of Otolaryngology-Head and Neck Surgery, Johns Hopkins University School of Medicine, Baltimore, MD USA

**Keywords:** Particulate matter, PM2.5, Air pollution, Thyroid cancer, Papillary thyroid cancer, Socioeconomic disparities

## Abstract

**Background:**

Limited data exists suggesting that cumulative exposure to air pollution in the form of fine particulate matter (aerodynamic diameter ≤ 2.5 μm [PM_2.5_]) may be associated with papillary thyroid carcinoma (PTC), although this relationship has not been widely established. This study aims to evaluate the association between PM_2.5_ and PTC and determine the subgroups of patients who are at the highest risk of PTC diagnosis.

**Methods:**

Under IRB approval, we conducted a case-control study of adult patients (age ≥ 18) newly diagnosed with PTC between 1/2013–12/2016 across a single health care system were identified using electronic medical records. These patients were compared to a control group of patients without any evidence of thyroid disease. Cumulative PM_2.5_ exposure was calculated for each patient using a deep learning neural networks model, which incorporated meteorological and satellite-based measurements at the patients’ residential zip code. Adjusted multivariate logistic regression was used to quantify the association between cumulative PM_2.5_ exposure and PTC diagnosis. We tested whether this association differed by gender, race, BMI, smoking history, current alcohol use, and median household income.

**Results:**

A cohort of 1990 patients with PTC and a control group of 6919 patients without thyroid disease were identified. Compared to the control group, patients with PTC were more likely to be older (51.2 vs. 48.8 years), female (75.5% vs 46.8%), White (75.2% vs. 61.6%), and never smokers (71.1% vs. 58.4%) (*p* < 0.001). After adjusting for age, sex, race, BMI, current alcohol use, median household income, current smoking status, hypertension, diabetes, COPD, and asthma, 3-year cumulative PM_2.5_ exposure was associated with a 1.41-fold increased odds of PTC diagnosis (95%CI: 1.23–1.62). This association varied by median household income (p-interaction =0.03). Compared to those with a median annual household income <$50,000, patients with a median annual household income between $50,000 and < $100,000 had a 43% increased risk of PTC diagnosis (aOR = 1.43, 95%CI: 1.19–1.72), and patients with median household income ≥$100,000 had a 77% increased risk of PTC diagnosis (aOR = 1.77, 95%CI: 1.37–2.29).

**Conclusions:**

Cumulative exposure to PM_2.5_ over 3 years was significantly associated with the diagnosis of PTC. This association was most pronounced in those with a high median household income, suggesting a difference in access to care among socioeconomic groups.

**Supplementary Information:**

The online version contains supplementary material available at 10.1186/s12940-023-00972-1.

## Introduction

Thyroid cancer incidence has been rising in the United States since the 1970s [1]. Although many new cancer diagnoses may be attributed to the increased detection of occult tumors, the steady rise in the incidence of larger tumors and regional disease suggests that other factors may play a role in this increase [1]. Established risk factors for thyroid cancer include female gender, racial/ethnic minority status, obesity, and low socioeconomic status [1–4]. Somewhat paradoxically, several studies have shown that alcohol consumption and tobacco use may decrease the risk of thyroid cancer [5–7]. However, the magnitude of increased thyroid cancer incidence varies substantially worldwide, leading prior investigators to examine the role of both indoor and outdoor environmental pollutants including agricultural fertilizers, organic pesticides, bisphenols, and phthalates in thyroid carcinogenesis [8–13]. Furthermore, a greater incidence of thyroid cancer has also been observed in areas with high volcanic activity, due either to carcinogenic activity of heavy metal contamination in the soil or exposure to increased levels of air pollution [14, 15].

Air pollution consists of gases and particulate matter, a complex mixture of solid and liquid particles that become suspended in the air following combustion and are classified by aerodynamic diameter [16]. The Environmental Protection Agency (EPA) monitors two size ranges of particulate matter: particles that are ≤10 μm in diameter (PM_10_) and particles that are ≤2.5 μm in diameter (PM_2.5_) [17]. PM_2.5_ is finer and can penetrate the lung barrier and enter the bloodstream, and thus it is believed to be the most pathogenic component of air pollution [11]. In 2013, the World Health Organization declared particulate matter air pollution carcinogenic [18]. Recent studies from South Korea, China, and Brazil have suggested that ambient particulate matter air pollution may be associated with thyroid cancer [19–22]. Additionally, observational studies have noted an increase in the rate of thyroid cancer among workers who participated in the rescue and recovery efforts at the World Trade Center following the terrorist attacks of September 11th, 2001 [23, 24]. These workers, who were exposed to PM_2.5_ levels over seven times higher than current acceptable EPA standards, have a two-fold greater incidence of thyroid cancer compared to the general population [25, 26]. In the United States, we recently performed a study suggesting an association between rising concentrations of PM_2.5_ and the clinical diagnosis of PTC [27, 28]. Thus, limited but consistent data exists regarding the association between PM_2.5_ and thyroid cancer diagnosis.

However, the burden of air pollution is not shared equally. Around 41% of people in the United States live in areas with unhealthy air quality levels [29]. Compared to healthy adults, vulnerable populations such as children, older adults, and those living with chronic conditions may be more susceptible to the health effects of air pollution [29]. Furthermore, prior research has demonstrated that those of lower socioeconomic status are more likely to be exposed to higher levels of air pollution during their lifetime [30]. Reasons for such a disparity may include the increased use of public transportation, housing location next to high-pollutant areas such as highways and industrial plants (the presence of which typically devalues the land, making it more accessible to those with a low socioeconomic status), higher rates of occupational pollutant exposure, and building type and age of the home in which the individual resides [30, 31]. Access to healthcare can also influence overall health status.

However, little is known about whether these vulnerable populations are more likely to be diagnosed with PTC when exposed to air pollution. Therefore, the purpose of this study was to more closely assess the association between PM_2.5_ exposure and PTC diagnosis and determine those who are most likely to be diagnosed with PTC.

## Methods

### Study population and design

**Fig. 1:**
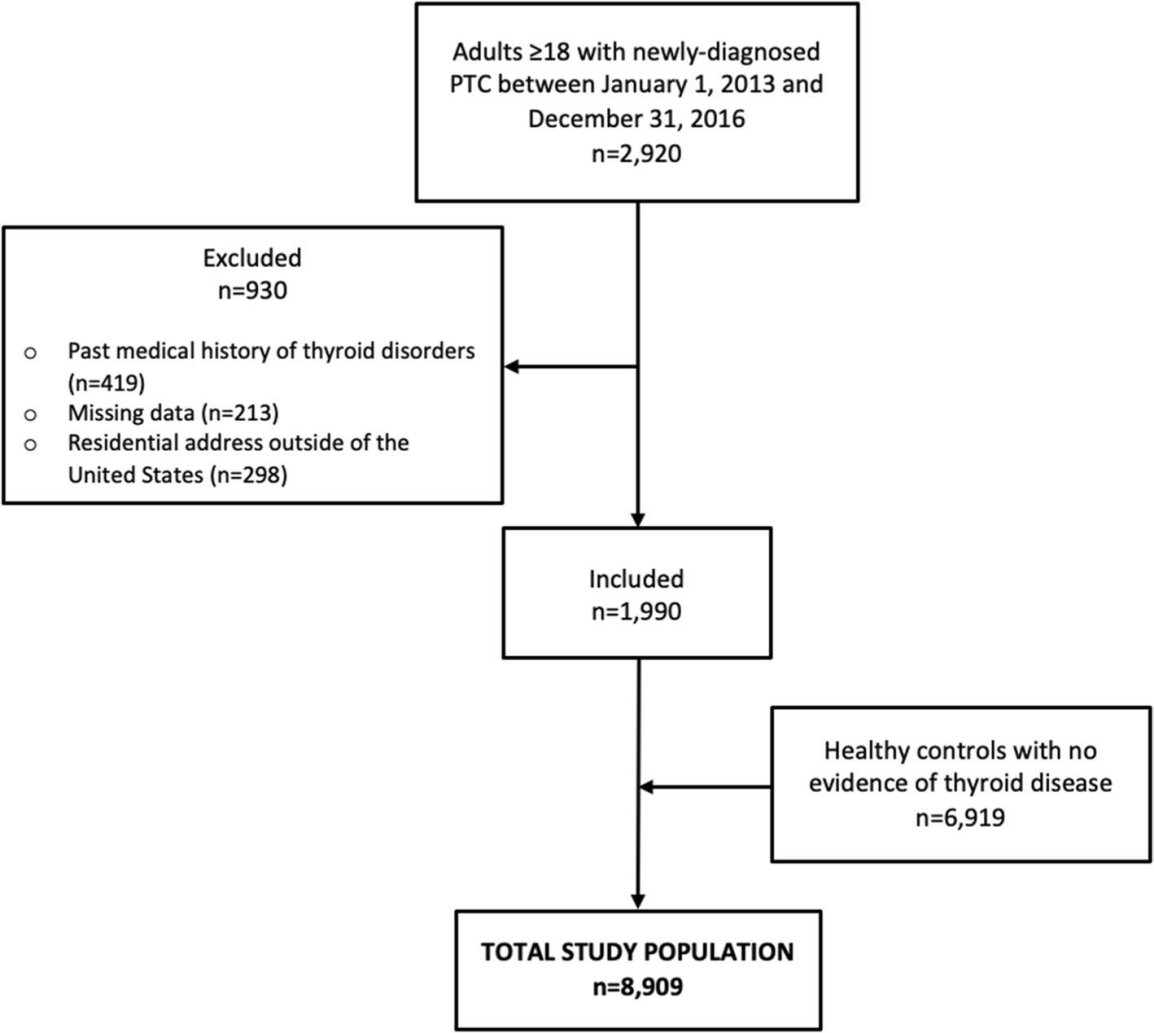
Inclusion and exclusion criteria for the study population

Under IRB approval, we performed a case-control study utilizing a large-volume electronic medical record data extract using clinical, demographic, and radiographic records from the Johns Hopkins Medical Institution in the United States. We identified 2920 adult patients (≥18 years old) with newly diagnosed PTC between January 1, 2013 and December 31, 2016. PTC was selected as it is the most common subtype of thyroid cancer and is primarily responsible for the increased incidence of thyroid cancer in the last several decades [1]. To ensure that we did not include patients who may have had occult thyroid cancer that had gone undiagnosed prior to the study period, we excluded patients with a past medical history of thyroid disorders (ICD-10 E07.9), thyroid cancer (ICD-10 C73), or thyroid nodules (ICD-10 E04.1) (*n* = 419). In addition, we excluded patients with missing data (*n* = 213) and those residing outside of the United States (*n* = 298) for a final sample size of 1990 patients. Using the same medical record extraction, we compared this cohort of PTC patients to a control group of 6919 healthy patients without any evidence of thyroid cancer or thyroid nodules based on computed tomography (CT) or magnetic resonance imaging (MRI) and who did not have a diagnosis of hyperthyroidism or hypothyroidism. This resulted in a total study population of 8909 patients (Fig. 1).

Patient characteristics including age, sex, race, body mass index (BMI), smoking status, and alcohol consumption were extracted from medical records. Additionally, the presence of comorbidities including hypertension, diabetes, COPD, asthma, nasal polyps, and environmental allergies were also obtained from medical records. Median household income was determined using data from the US Census Bureau’s American Community Survey, which collects data regarding annual income from 3.5 million households each year [32]. This data is stratified by geographic location based on the household’s 5-digit zip code in order to determine the median annual household income for each zip code in the country [33]. We used this information to estimate the annual household income for each individual in the study based on their zip code of residence at the time they were diagnosed with PTC. Median annual household income was adjusted for inflation to match 2016 US dollars.

### PM_2.5_ exposure assessment

Cumulative PM_2.5_ exposure prior to PTC diagnosis was calculated for each patient using a validated, deep learning neural networks model which incorporated air pollution monitoring data acquired from the US EPA Air Quality System as well as meteorological- and satellite-based measurements, including temperature, humidity, dew point, wind speed, pressure, population density, sea level, and land-use information. These data were used to estimate the PM_2.5_ exposure level for every 3 km × 3 km square grid throughout the continental United States. Cumulative PM_2.5_ exposure was then determined according to the zip code of each patients’ address of residence at the time of diagnosis of PTC or at a matched time for those in the control group. For example, if a patient was diagnosed with PTC in 2015, then that patient’s cumulative PM_2.5_ exposure over 36 months was determined by calculating the total PM_2.5_ levels between 2012 and 2015 according to the zip code in which the patient lived in 2015.

Holdout cross-validation and 10-fold cross-validation were used to evaluate model performance [34]. For holdout cross-validation, we randomly selected 70% of the data from each region to fit the model and held out 30% of the data for validation. For 10-fold cross-validation, we used 90% randomly selected data to train the models, and 10% of the data was held out for validation in 10 separate iterations. Cross-validation indicated that the models had a high predictive accuracy across the entire study area; the coefficient of determination (R^2^) for PM_2.5_ was 0.86, with a variation between 0.71 and 0.93 and the mean square error between the measurements and predicted values for PM_2.5_ was 1.50 mg/m^3^. A variety of exposure metrics were created as appropriate to examine different potential important periods of exposure, including 12-, 24-, and 36-month mean PM_2.5_ concentration before the diagnosis date. Cumulative exposure to PM_2.5_ for each year was determined using a 365-day moving average of air pollutant concentrations between the diagnosis day and the pre-364th day. This model has been used previously to study the impact of PM_2.5_ levels on arrhythmias and depression in Asia [35, 36]. It has additionally been used in a study in the United States evaluating the relationship between PM_2.5_ and the development of anosmia [37].

### Statistical analysis

Descriptive analyses were performed using mean (standard deviation) or frequency (percentage). Statistical significance was calculated using a Chi-squared test or a Mann-Whitney U test for categorical variables and a t-test for continuous variables.

The association between diagnosis of PTC and short-term cumulative PM_2.5_ exposure over 12, 24, and 36 months of was estimated using logistic regression models. Three different models were used to evaluate this association, consistent with previous studies evaluating the association between PM_2.5_ and lung cancer or cardiovascular disease [38–40]. Model 1 adjusted demographic characteristics including age, sex, race, and state of residence. Model 2 was further adjusted for risk factors for thyroid cancer including BMI, current alcohol use, median household income, smoking status, and alcohol use. Model 3 was further adjusted for comorbidities that may confound this association including hypertension, diabetes, COPD, and asthma. A Wald test for interaction was then performed to determine whether the association between diagnosis of PTC and cumulative exposure to PM_2.5_ over 36 months differed by potential risk factors for PTC including sex, race (White, Black, Hispanic/Latino, or other), BMI (< 18.5 kg/m^2^, 18.5 to < 25 kg/m^2^, 25 to < 30 kg/m^2^, or ≥ 30 kg/m^2^), smoking status (never smoker, current smoker, or former smoker), alcohol consumption (current drinker or never drinker), and median annual household income (<$50,000, $50,000 to <$100,000, and ≥ $100,000). These associations were reported as adjusted odds ratios (aORs) with associated 95% confidence intervals (CIs).

Statistical analyses were conducted using STATA, version 16.0 (Stata Corporation, College Station, TX) and R, version 4.1 (R Development Core Team, Vienna, Austria). Two-sided *p*-values < 0.05 were considered statistically significant.

### Sensitivity analysis

A sensitivity analysis was performed in which PTC patients and controls were matched by age, gender, race/ethnicity, and BMI in a 1:1, 1:2, and 1:3 matching ratio using the nearest neighbor matching method. The association between diagnosis of PTC and cumulative PM_2.5_ concentrations over 12, 24, and 36 months of exposure was estimated using logistic regression models.

## Results

### Characteristics of the study population

**Fig. 2:**
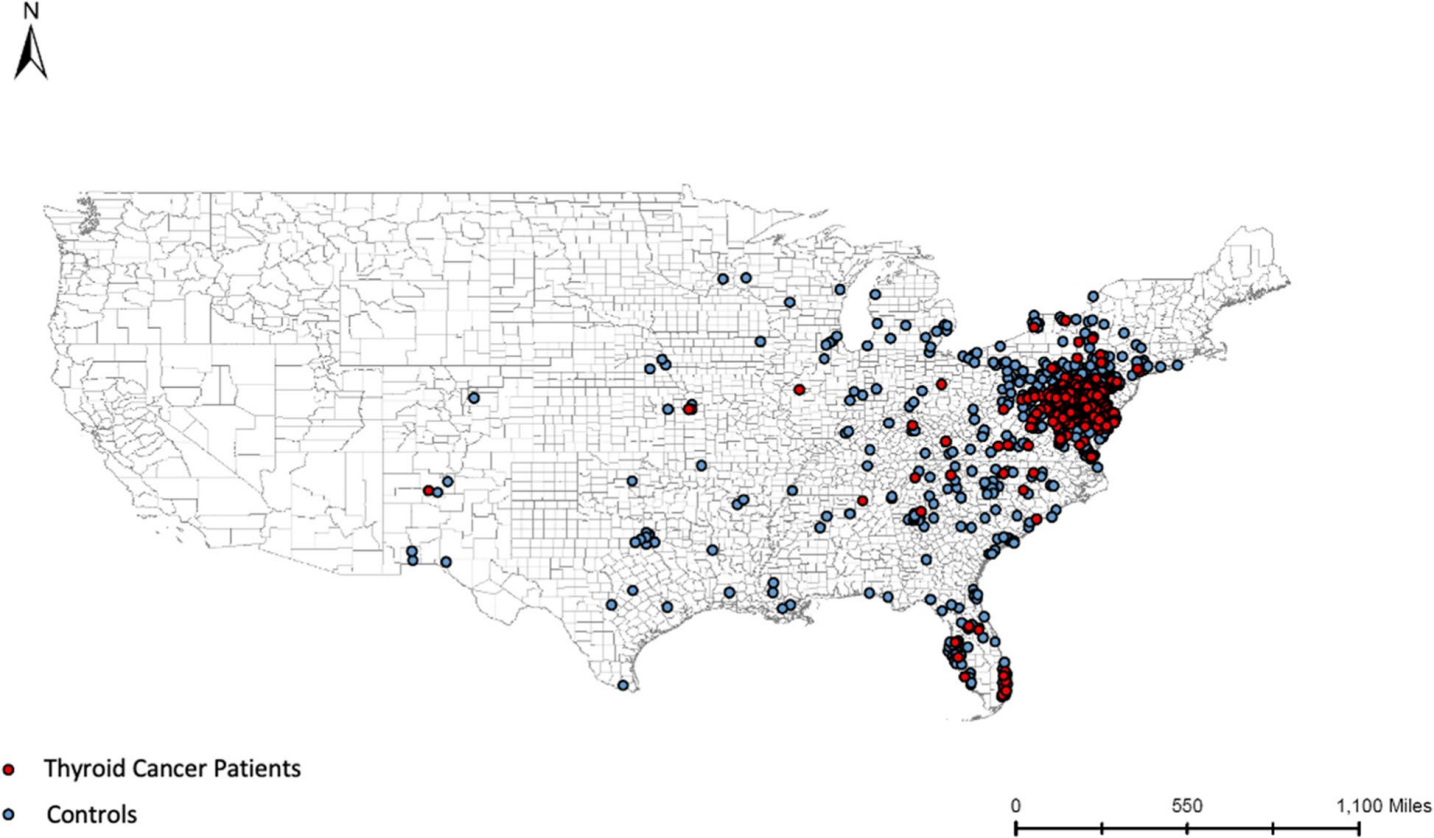
Geographic distribution of study patients

A total of 1990 patients with newly diagnosed PTC were compared to a control group of 6919 patients without thyroid disorders (Table 1). Compared to the control group, patients with PTC were more likely to be older, female sex, White race, and have a BMI ≥30 kg/m^2^ (all *p* < 0.001). In addition, patients with PTC were more likely to be non-smokers and non-alcohol consumers (all *p* < 0.001). Patients with PTC also had a higher median household income. Hypertension, diabetes mellitus, COPD, asthma, nasal polyps, and environmental allergies were more prevalent among the control subjects when compared to patients with PTC (p < 0.001). Patients mostly resided in the East coast of the United States (Fig. 2).

**Table 1:** Clinical and demographic patient characteristics by cases of newly diagnosed papillary thyroid cancer (PTC) and healthy controls without any thyroid disorders

Characteristics	Controls (***n*** = 6919)	PTC (***n*** = 1990)	***p*** - Value
**Age (y), mean (SD)**	48.84 (17.76)	51.19 (14.93)	< 0.001
**Male sex, %**	3678 (53.2)	487 (24.5)	< 0.001
**Race, %**			< 0.001
White	4262 (61.6)	1496 (75.2)	
African American	1935 (28.0)	189 (9.5)	
Hispanic /Latino ethnicity	250 (3.6)	139 (7.0)	
Other	472 (6.8)	166 (8.3)	
**Cumulative average PM**_**2.5**_ **(μg/m**^**3**^**), mean (SD)**			
12-months	10.09 (1.88)	10.00 (1.77)	0.05
24-months	10.34 (1.92)	10.31 (1.89)	0.51
36-months	10.59 (2.00)	10.61 (1.97)	0.74
**Body mass index (kg/m**^**2**^**), %**			< 0.001
< 18.5	234 (3.4)	22 (1.1)	
18.5 to < 25	2460 (35.6)	664 (33.4)	
25 to < 30	2190 (31.7)	672 (33.8)	
≥ 30	2035 (29.4)	632 (31.8)	
**Smoking status, %**			< 0.001
Never smoker	4042 (58.4)	1414 (71.1)	
Current smoker	883 (12.8)	104 (5.2)	
Former smoker	1994 (28.8)	472 (23.7)	
**Current alcohol consumption**	2904 (42.0)	699 (35.1)	< 0.001
**Median annual household income (US $)**^**a**^**, %**			< 0.001
< 50,000	1592 (23.0)	194 (9.7)	
50,000 to < 100,000	3992 (57.7)	1098 (55.2)	
≥ 100,000	1335 (19.3)	698 (35.1)	
**Comorbidities (%)**			
Hypertension	1995 (28.8)	310 (15.6)	< 0.001
Diabetes mellitus	668 (9.7)	108 (5.4)	< 0.001
COPD	190 (2.7)	12 (0.6)	< 0.001
Asthma	795 (11.5)	76 (3.8)	< 0.001
Nasal Polyps	155 (2.2)	0 (0.0)	< 0.001
Environmental allergy	178 (2.6)	16 (0.8)	< 0.001

*PM*_*2.5*_ fine (diameter < 2.5 μm) particulate matter, *COPD* Chronic obstructive pulmonary disease

^a^Inflation-adjusted to match 2016 US dollars

### Association between PM_2.5_ exposure and incident PTC diagnoses

After adjusting for age, sex, race, BMI, current alcohol use, median household income, smoking status, alcohol use, hypertension, diabetes, COPD, and asthma, a 10 μg/m^3^ increase in cumulative PM_2.5_ concentration over 12 months was significantly associated with the incident diagnosis of PTC (aOR = 1.27, 95% CI: 1.07–1.52). Additionally, the likelihood of PTC diagnosis was greater with increasing cumulative exposure to PM_2.5_ over 24 months (aOR = 1.36, 95% CI: 1.15–1.61) and 36 months (aOR = 1.42, 95% CI: 1.21–1.67) (Table 2).

**Table 2:** Association between cumulative exposure to fine (diameter ≤ 2.5 μm) particulate matter (PM_2.5_) and diagnosis of papillary thyroid cancer (PTC)

Cumulative exposure to PM_**2.5**_	PTC diagnosis aOR (95%CI)		
	Model 1	Model 2	Model 3
12 months	1.24 (1.05, 1.47)	1.26 (1.06, 1.50)	1.27 (1.07, 1.52)
24 months	1.36 (1.15, 1.60)	1.36 (1.15, 1.60)	1.36 (1.15, 1.61)
36 months	1.43 (1.22, 1.67)	1.42 (1.21, 1.67)	1.42 (1.21, 1.67)

Model 1: Adjusted for age, sex, race, and state of residence

Model 2: Model 1 + BMI, current alcohol use, median household income, current smoking status

Model 3: Model 2 + hypertension, diabetes, COPD, and asthma

*aOR* Adjusted odds ratio, *CI* Confidence interval

These finding were consistent in the sensitivity analysis when patients were matched by age, gender, race/ethnicity, and BMI at a 1:1, 1:2, and 1:3 matching ratios (Supplemental Table S1).

### PTC incidence among subgroups

When stratified by various patient subgroups, the association between cumulative exposure to PM_2.5_ over 36 months and PTC diagnosis differed only by median annual household income (p_interaction_ = 0.03) (Table 3). For those with a median annual household income of <$50,000, cumulative exposure to PM_2.5_ was not associated with an increased incidence of PTC (aOR = 0.99, 95% CI: 0.71–1.40). However, increased exposure to PM_2.5_ was associated with a higher likelihood of being diagnosed with PTC among those with a median annual household income between $50,000 and $100,000 as well as >$100,000 (aOR = 1.43, 95% CI: 1.19–1.72 and OR = 1.77, 95% CI: 1.37–2.29, respectively).

**Table 3:** Association between cumulative exposure to fine (diameter ≤ 2.5 μm) particulate matter (PM_2.5_) over 36 months and diagnosis of papillary thyroid cancer (PTC) by various patient characteristics. Models were adjusted for age, sex, race, BMI, current alcohol use, median household income, current smoking status, hypertension, diabetes, COPD, and asthma

Patient Characteristics	n	PTC Diagnosis aOR (95% CI)	p-value
**Sex**			0.64
Male	4744	1.47 (1.23, 1.75)	
Female	4165	1.38 (1.10, 1.72)	
**Race**			0.32
White	5758	1.42 (1.22, 1.66)	
African American	2124	1.71 (1.05, 2.79)	
Hispanic/Latino	389	1.91 (1.08, 3.37)	
Other	638	1.07 (0.66, 1.75)	
**BMI**			0.74
Underweight, < 18.5	256	4.54 (1.24, 16.62)	
Normal weight, 18.5 to < 25	3124	1.49 (1.19, 1.86)	
Overweight, 25 to < 30	2862	1.43 (1.14, 1.81)	
Obesity, > = 30	2667	1.28 (1.00, 1.69)	
**Smoking status**			0.11
Never smoker	5456	1.54 (1.30, 1.80)	
Current smoker	987	0.83 (0.47, 1.48)	
Former smoker	2466	1.33 (1.00, 1.76)	
**Alcohol consumption**			0.46
Never drinker	5306	1.37 (1.16, 1.64)	
Current drinker	3603	1.53 (1.23, 1.91)	
**Median annual household income (US $)**^**a**^			**0.03**
< 50,000	1786	0.99 (0.71, 1.40)	
50,000 to < 100,000	5090	**1.43 (1.19, 1.72)**	
> =100,000	2033	**1.77 (1.37, 2.29)**	

Associations that are statistically significant at *p* < 0.05 are bolded

*aOR* Adjusted odds ratio, *CI* Confidence interval

^a^Inflation-adjusted to match 2016 US dollars

The association between cumulative PM_2.5_ exposure over 36 months and PTC diagnosis did not vary by sex, race/ethnicity, BMI, smoking status, or current alcohol consumption (all p_interaction_ > 0.05) (Table 3).

These findings were similar when analyzing cumulative PM_2.5_ exposure over 12 months and 24 months (Supplemental Table S2 and S3).

## Discussion

This study of 8909 patients is the first in the US to demonstrate the impact of socioeconomic status on the association between PM_2.5_ exposure and incident PTC diagnoses. A 10 μg/m^3^ increase in PM_2.5_ concentration over 12, 24, and 36 months was associated with a greater likelihood of being diagnosed with PTC, with this likelihood increasing with increasing duration of exposure to PM_2.5_. When stratified by median annual household income, those with a higher median annual income are more likely to be diagnosed with PTC. Our study highlights the socioeconomic disparities seen in PTC diagnosis in the United States.

Similar to our study, others have investigated the impact of ambient air pollution on thyroid cancer. A study investigating the impact of PM on cancer incidence and mortality in São Paulo, Brazil found that levels of PM_10_ correlated significantly with the incidence of thyroid cancer [22]. Additionally, a study of 550,000 patients in China showed that emissions from industrial waste gas, made up mostly of particulate matter, sulfur dioxide, and nitrogen dioxide, were associated with increased rates of several malignancies, including thyroid cancer [20]. Several air pollutants, including PM_2.5_, have been associated with an increased risk for the development of thyroid nodules, although it is unclear if this correlates with a greater incidence of thyroid cancer [41]. Our study built upon these findings by showing an association between PM_2.5_ and PTC diagnosis, and demonstrating that the risk of PTC diagnosis increases with increasing duration of exposure to PM_2.5_.

In contrast to our study, Park et al. found that exposure to particulate matter was negatively associated with the incidence of thyroid cancer [19]. However, this study did not discuss the methods used to measure levels of particulate matter. Furthermore, it was not clear how these levels were linked to each patient, limiting the generalizability of these findings.

Additionally, Giannoula et al. used pollution data from 27 European countries and found that the air pollutants Benzo (k) Fluoranthene and HexaChlorocycloHexane positively correlated with the incidence of thyroid cancer in men [42]. Unlike our study, they did not find that relationship between these pollutants and thyroid cancer diagnosis varied by socioeconomic status. However, socioeconomic status in this study was determined by the Gross National Income per capita for the country in which each patient lived, which may be too broad of an index to appropriately assess the true socioeconomic status of each individual patient.

The mechanism for the association between PM_2.5_ and thyroid cancer is unclear. This association may be related to volatile organic compounds (VOCs) which are often bound to PM and can penetrate the alveolar barrier entering the bloodstream [43]. VOCs may act as endocrine disrupting chemicals when entering the body in this manner, interacting with thyroid hormone receptors and altering thyroid homeostasis [43]. It is also possible that pollution induces epigenetic DNA alterations leading to neoplastic changes [44]. However, this mechanism is not completely understood, and further research is needed to better determine the role that PM plays in the development thyroid cancer.

The relationship between socioeconomic status and incidence of thyroid cancer has been described previously. Worldwide, the incidence of thyroid cancer is 4- to 5-fold higher in developed countries when compared to developing countries [45]. Within the United States, both the overall incidence of thyroid cancer as well as the rate at which this incidence is increasing is greater in areas with a higher socioeconomic status, determined by both annual income and level of education [3, 46]. Conversely, low socioeconomic status has been associated with a more advanced stage of thyroid cancer at the time of presentation [47], as well as worse disease-specific survival [48]. The reason for this association may be related to differences in access to healthcare. The risk of PTC diagnosis among those who have insurance, a marker of socioeconomic status, is 2.5-fold higher when compared to the uninsured [49]. It is possible that those with a higher socioeconomic status visit healthcare providers more frequently, which may enable increased imaging and biopsy of suspicious thyroid nodules, explaining the higher rates of diagnosis among this demographic. This is particularly evident among patients with smaller, asymptomatic thyroid nodules that may otherwise go undiagnosed, as the socioeconomic disparity in thyroid cancer diagnosis disappears in patients with nodules ≥4 cm [3]. While our study did not capture tumor size, we were nevertheless able to add to these findings by showing that the socioeconomic disparity in the incidence of PTC persists even when accounting for exposure to PM_2.5_. These results suggest a difference in access to healthcare among various socioeconomic groups.

The strengths of this study include the large study population and the comprehensive clinical and demographic information. Air pollution monitoring models were also highly accurate, which allowed us to confidently predict the cumulative exposure to ambient PM_2.5_ at the level of each individual’s zip code. These models also allowed for more accurate PM_2.5_ prediction for areas in which EPA monitors are lacking.

There are several limitations to this study, including those inherent to retrospective data collection with large EMR extracts such as human error in data collection, misclassification, and missing values. We were unable to account for certain risk factors for thyroid cancer, such as family history or history of ionizing radiation, as this information is not reliably available in large electronic medical record abstractions. We were also unable to identify the level of education or the occupational status for each patient, which may be a confounder in the association between PTC diagnosis and socioeconomic status. Annual income for each patient is also only an estimate based on the median household income of the zip code of residence, and therefore may not reflect each individual’s actual annual income. This study may have also been subject to measurement bias, as the spatial accuracy of a zip code is limited. Additionally, we could not account for social migration for each individual in the study. In order to calculate the cumulative exposure to PM_2.5_ over 3 years, our models assumed that the zip code in which the patient resided at the time of diagnosis of PTC was the same zip code in which they lived throughout the study period. It is possible that some patients resided in multiple zip codes throughout the study period, giving them varying levels of PM_2.5_ exposure. Unfortunately, contact information for each patient was not available in the dataset provided, and as such we were unable to survey each individual regarding where they lived during the study period. These models also did not account for levels of indoor air pollution, occupational exposure to pollutants, or ingestion of potential toxins, each of which may have impacted the overall level of pollution that each individual was exposed to.

It is also difficult to know for certain how long a cancer has been present prior to diagnosis, as small asymptomatic cancers could have gone undetected for several years, although this was mitigated somewhat as patients with a previous history of thyroid nodules or other thyroid disorders were excluded from the study. Similarly, information regarding tumor size, nodal status, and pathologic features of each tumor were not available in the data extract, and therefore could not be utilized to determine if the disease was diagnosed at more advance stages. While some studies have shown that thyroid cancer may develop as early as 3 years following certain environmental insults [50], cumulative exposure of PM_2.5_ up to 36 months prior to diagnosis may be too short of a time period to capture the full impact of PM_2.5_ on the incidence of PTC. Unfortunately, the electronic medical record database used for the record extraction in this study did not become fully functional until 2013, and so it was not possible to calculate PM_2.5_ for individuals prior to this time. Finally, while patients in this study were distributed throughout the United States, the majority resided in the East coast, and therefore the study population might not be entirely representative of those living in other parts of country and worldwide.

## Conclusions

Cumulative exposure to ambient air pollution in the form of PM_2.5_ is associated with the incident diagnosis of PTC. The likelihood of PTC diagnosis increases with increasing duration of exposure to PM_2.5_. Among those exposed to PM_2.5_, individuals with a higher median annual income are more likely to be diagnosed with PTC, suggesting a socioeconomic disparity in the diagnosis of thyroid cancer. This study provides further evidence for the negative health effects of PM_2.5_. These findings shed light on current EPA standards for acceptable PM_2.5_ levels and question whether those standards should be lowered in order to reduce the risk of PTC. However, further research regarding the association between PM_2.5_ and PTC needs to be done nationally in order to influence policy decision makers.

## Supplementary Information

**Additional file 1: Supplemental Table S1.** Sensitivity analysis for the association between cumulative exposure to fine (diameter ≤2.5 μm) particulate matter (PM_2.5_) and diagnosis of papillary thyroid cancer (PTC). Patients were matched by age, gender, race/ethnicity, and BMI using a 1:1, 1:2, and 1:3 matching ratio.

**Additional file 2: Supplemental Table S2.** Association between cumulative exposure to fine (diameter ≤2.5 μm) particulate matter (PM_2.5_) over 12 months and diagnosis of papillary thyroid cancer (PTC) by various patient characteristics. Models were adjusted for age, sex, race, BMI, current alcohol use, median household income, current smoking status, hypertension, diabetes, COPD, and asthma.

**Additional file 3: Supplemental Table S3.** Association between cumulative exposure to fine (diameter ≤ 2.5 μm) particulate matter (PM_2.5_) over 24 months and diagnosis of papillary thyroid cancer (PTC) by various patient characteristics. Models were adjusted for age, sex, race, BMI, current alcohol use, median household income, current smoking status, hypertension, diabetes, COPD, and asthma.

## Data Availability

The datasets used and/or analyzed during the current study are available from the corresponding author on reasonable request.

## Abbreviations

CI: Confidence interval
EPA: Environmental Protection Agency
OR: Odds ratio
PM: Particulate matter
PM_2.5_: Fine (diameter ≤ 2.5 μm) particulate matter
PM_10_: Fine (diameter ≤ 10 μm) particulate matter
PTC: Papillary thyroid cancer
VOC: Volatile organic compound
