# Supplementary material for: Socioeconomic disparity in the association between fine particulate matter exposure and papillary thyroid cancer
Source: Environ Health. 2023 Feb 23;22:20. doi: 10.1186/s12940-023-00972-1 (PMC9948306; doi:10.1186/s12940-023-00972-1)
Supplement: Supplementary file 1 — Additional file 1: Supplemental Table S1. Sensitivity analysis for the association between cumulative exposure to fine (diameter ≤2.5 μm) particulate matter (PM2.5) and diagnosis of papillary thyroid cancer (PTC). Patients were matched by age, gender, race/ethnicity, and BMI using a 1:1, 1:2, and 1:3 matching ratio. [file 12940_2023_972_MOESM1_ESM.docx]

**Supplemental Table S1.** Sensitivity analysis for the association between cumulative exposure to fine (diameter ≤2.5 μm) particulate matter (PM_2.5_) and diagnosis of papillary thyroid cancer (PTC). Patients were matched by age, gender, race/ethnicity, and BMI using a 1:1, 1:2, and 1:3 matching ratio.

| **Cumulative exposure to PM_2.5_** | **PTC diagnosis**  aOR (95% CI) |
| --- | --- |
| **1:1 Matching Ratio** |  |
| 12 months | 1.28 (0.89, 1.83) |
| 24 months | 1.32 (0.94, 1.85) |
| 36 months | 1.38 (0.99, 1.92) |
| **1:2 Matching Ratio** |  |
| 12 months | 1.24 (0.96, 1.60) |
| 24 months | 1.34 (1.05, 1.71) |
| 36 months | 1.41 (1.11, 1.79) |
| **1:3 Matching Ratio** |  |
| 12 months | 1.21 (1.00, 1.46) |
| 24 months | 1.27 (1.06, 1.53) |
| 36 months | 1.32 (1.11, 1.58) |
| Models were adjusted for age, sex, race, BMI, current alcohol use, median household income, current smoking status, hypertension, diabetes, COPD, and asthma.  aOR = adjusted odds ratio; CI = confidence interval | |
