# Supplementary material for: Socioeconomic disparity in the association between fine particulate matter exposure and papillary thyroid cancer
Source: Environ Health. 2023 Feb 23;22:20. doi: 10.1186/s12940-023-00972-1 (PMC9948306; doi:10.1186/s12940-023-00972-1)
Supplement: Supplementary file 2 — Additional file 2: Supplemental Table S2. Association between cumulative exposure to fine (diameter ≤2.5 μm) particulate matter (PM2.5) over 12 months and diagnosis of papillary thyroid cancer (PTC) by various patient characteristics. Models were adjusted for age, sex, race, BMI, current alcohol use, median household income, current smoking status, hypertension, diabetes, COPD, and asthma. [file 12940_2023_972_MOESM2_ESM.docx]

| **Supplemental Table S2.** Association between cumulative exposure to fine (diameter ≤2.5 μm) particulate matter (PM_2.5_) over **12 months** and diagnosis of papillary thyroid cancer (PTC) by various patient characteristics. Models were adjusted for age, sex, race, BMI, current alcohol use, median household income, current smoking status, hypertension, diabetes, COPD, and asthma. | | | |
| --- | --- | --- | --- |
| **Patient Characteristics** | **N** | **PTC Diagnosis**  aOR (95% CI) | **p-value** |
| **Sex** |  |  | 0.69 |
| Male | 4,744 | 1.32 (1.09, 1.60) |  |
| Female | 4,165 | 1.25 (0.99, 1.57) |  |
| **Race** |  |  | 0.12 |
| White | 5758 | 1.29 (1.09, 1.53) |  |
| African American | 2124 | 1.71 (1.00, 2.91) |  |
| Hispanic/Latino | 389 | 1.77 (0.97, 3.23) |  |
| Other | 638 | 0.78 (0.45, 1.35) |  |
| **BMI** |  |  | 0.60 |
| Underweight, <18.5 | 256 | 2.94 (1.17, 20.9) |  |
| Normal weight, 18.5 to <25 | 3124 | 1.29 (1.03, 1.63) |  |
| Overweight, 25 to <30 | 2862 | 1.37 (1.06, 1.77) |  |
| Obesity, >=30 | 2667 | 1.12 (0.84, 1.50) |  |
| **Smoking status** |  |  | 0.09 |
| Never smoker | 5456 | 1.38 (1.16, 1.65) |  |
| Current smoker | 987 | 0.67 (0.36, 1.25) |  |
| Former smoker | 2466 | 1.25 (0.91, 1.71) |  |
| **Alcohol consumption** |  |  | 0.41 |
| Never drinker | 5306 | 1.23 (1.02, 1.48) |  |
| Current drinker | 3603 | 1.39 (1.10, 1.77) |  |
| **Median annual household income (US $)^a^** |  |  | 0.04 |
| < 50,000 | 1,786 | 0.88 (0.62, 1.27) |  |
| 50,000 to < 100,000 | 5,090 | **1.29 (1.06, 1.58)** |  |
| >=100,000 | 2,033 | **1.60 (1.22, 2.12)** |  |
| Associations that are statistically significant at p<0.05 are bolded.  aOR = adjusted odds ratio; CI = confidence interval  ^a^Inflation-adjusted to match 2016 US dollars. | | | |
